# Hospital discharge processes: Insights from patients, caregivers, and staff in an Australian healthcare setting

**DOI:** 10.1371/journal.pone.0308042

**Published:** 2024-09-19

**Authors:** Olumuyiwa Omonaiye, Kristel Ward-Stockham, Peteris Darzins, Clinton Kitt, Evan Newnham, Nicholas F Taylor, Julie Considine

**Affiliations:** 1 Geelong: School of Nursing and Midwifery and Centre for Quality and Patient Safety in the Institute for Health Transformation, Deakin University, Geelong, Victoria, Australia; 2 Centre for Quality and Patient Safety–Eastern Health Partnership, Eastern Health, Box Hill, Victoria, Australia; 3 Eastern Health Institute, Eastern Health, Box Hill, Victoria, Australia; 4 Eastern Health, Box Hill, Victoria, Australia; 5 Eastern Health Clinical School, Monash University, Clayton, Victoria, Australia; 6 La Trobe University, Bundoora, Victoria, Australia; University of Verona, ITALY

## Abstract

Hospital discharge is a pivotal point in healthcare delivery, impacting patient outcomes and resource utilisation. Ineffective discharge processes contribute to unplanned hospital readmissions. This study explored hospital discharge process from the perspectives of patients, caregivers, and healthcare staff. Qualitative data were collected through semi-structured interviews with adult patients being discharged home from a medical ward, their caregivers, and healthcare staff at an Australian hospital. Thematic analysis followed established guidelines for qualitative research. A total of 65 interviews and 21 structured observations were completed. There were three themes: i) Communication, ii) System Pressure, and iii) Continuing Care. The theme ‘Communication’ highlighted challenges and inconsistencies in notifying patients, caregivers, and staff about discharge plans, leading to patient stress and frustration. Information overload during discharge hindered patient comprehension and satisfaction. Staff identified communication gaps between teams, resulting in uncertainty regarding discharge logistics. The theme ‘System Pressure’ referred to pressure to discharge patients quickly to free hospital capacity occasionally, even in the face of inadequate service provision on weekends and out-of-hours. The ‘Continuing Care’ theme drew attention to gaps in patient understanding of follow-up appointments, underscoring the need for clearer post-discharge instructions. The lack of structured systems for tracking referrals and post-discharge care coordination was also highlighted, potentially leading to fragmented care. The findings resonate with international literature and the current emphasis in Australia on improving communication during care transitions. Furthermore, the study highlights the tension between patient-centred care and health service pressure for bed availability, resulting in perceptions of premature discharges and unplanned readmissions. It underscores the need for strengthening community-based support and systems for tracking referrals to improve care continuity. These findings have implications for patient experience and safety and suggest the need for targeted interventions to optimise the discharge process.

## Introduction

Today, Australia is home to 25 million people with over 300 different ancestries, who speak around 490 languages and observe a wide range of cultural and religious traditions [1]. Australia has a two-tier healthcare system, consisting of public and private sectors. The provision of universal healthcare in Australia involves three levels of government [2]: the Federal Government funds and indirectly supports inpatient and outpatient care through the Medicare Benefits Scheme and outpatient prescription medicine through the Pharmaceutical Benefits Scheme [2]. It also regulates private health insurance, pharmaceuticals, and therapeutic goods but has a limited role in direct service delivery. While the State Governments own and manage public hospitals, ambulances, public dental care, community health (primary and preventive care), and mental health care [2]. The Local Governments deliver community and home-based health and support, environmental health services such as waste disposal, water fluoridation [2].

Hospital discharge represents a critical juncture in the continuum of healthcare delivery, with profound implications for patient outcomes, care quality, and resource utilisation [3]. Thus, hospital discharge serves as a pivotal moment in a patient’s healthcare journey, marking the transition from the acute care setting to continued care, often in a community or home-based environment [4]. Ineffective discharge processes contribute significantly to unplanned hospital readmissions [5]. Patients discharged prematurely [6] or without adequate post-discharge support are more likely to experience complications that necessitate their unplanned return to the hospital [7, 8]. A timely and well-managed hospital discharge process improves the efficient allocation of healthcare resources, by increasing hospital bed availability, reducing hospital and emergency department overcrowding, and enhancing capacity to accommodate new patients requiring acute care [9].

Despite the importance, of the hospital discharge process, it remains fraught with challenges, and research gaps persist in understanding and optimising this critical healthcare transition. In Australia, some 21% of patients encountered care coordination issues and 41% documented deficiencies in hospital discharge planning [10].As healthcare systems worldwide grapple with the complexities of contemporary patient care, understanding the challenges and exploring solutions in the context of hospital discharge has become paramount. One barrier to improving the hospital discharge process is the lack of detailed understanding of its organisation, including its interdependencies and the performance-shaping factors that influence it [11]. Therefore, the aim of this study was to explore and describe the processes of hospital discharge for adult medical patients being discharged home from patient, caregivers and staff perspectives.

## Method

### Study design

This study was conducted using a qualitative descriptive approach [12] and is reported according to the Consolidated Criteria for Reporting Qualitative Research [13]. The Functional Resonance Analysis Method (FRAM), a five-step approach designed to analyse performance variability in complex systems [11] was used as a conceptual framework. The first three steps guided the study; including deciding the purpose of the FRAM analysis (hospital discharge), identifying the functions necessary for that work to be achieved and describing each function, and identifying and describing variability in the identified functions.

### Setting

This study was conducted at Eastern Health, in Melbourne, Australia, which encompasses 65 sites across 21 locations and delivers 1.3 million episodes of care annually [14]. Eastern Health operates six general medical wards within three acute care hospitals, with daily discharges from each ward ranging from one to six patients. For this study, we specifically chose one 27 bed general medical ward from a 155-bed outer metropolitan hospital that discharges six patients per day with an average inpatient length of stay of 4.6 days. The ward is staffed by ward-based nursing, allied health and pharmacy staff, whilst medical staff are largely ward-based with additional responsibility for management of boarders in other wards. There are daily multidisciplinary team (MDT) meetings on weekdays and physician-led medical ward rounds 7 days per week. Discharge planning huddles take place in the afternoon of each weekday. Communication of plans to patients occurs separate to the MDT meetings. A hybrid medical record is in use at the hospital: electronic medications, paper-based progress notes and observations and an electronic patient flow system. The ward selected was general medicine based on previous work [15] that showed general medicine had the highest number of unplanned readmissions. The ward is located in a health service with three acute care hospitals, all with the same governance structures and clinical processes for general medicine.

### Inclusion and exclusion criteria

Study ward patients identified as ready for discharge (by the nurse-in-charge, daily multidisciplinary meeting or electronic bed management system) on the day(s) of data collection, their caregivers, and all staff members from the study ward were eligible for inclusion. Patients who were discharged to destinations other than home, such as residential care, supported care, transitional care, or another hospital, were excluded. There were no exclusion criteria for staff.

### Sampling and recruitment of participants

Eligible patients without cognitive impairment and who were able to provide consent were informed about the study by nursing staff, provided with study information by the research team, and provided written informed consent for the interviews. Patients’ cognitive and clinical status was assessed by the patient’s treating team (medical and nursing staff): assessment of cognition is a usual clinical responsibility. The researchers checked the patients’ clinical notes (4AT delirium screen / Glasgow Coma Score documented by the clinical teams) and checked with the nurse in charge/ patient’s primary nurse before approaching the patient. Caregivers were interviewed either with the patient or separately, depending on patient preference or capacity; written informed consent was obtained before all interviews. Staff were informed about the study through emails and meetings. A subset of staff from various roles were purposively sampled for follow-up interviews to ensure representation.

### Ethical considerations

We obtained written informed consent from patients, and where applicable, caregivers to observe the discharge process. Additionally, all participants who were interviewed provided their written informed consent. The study was approved by the Human Research Ethics Committee at Eastern Health (LR21-019-73462) and Deakin University (2021–237).

### Data collection

From 8 November– 8 December 2022, semi-structured interviews were conducted by either by OO, a male doctoral-prepared public health researcher, or KWS, a master’s-prepared female nurse researcher. While KWS was acquainted with ward nursing staff, neither researcher had any prior connections with patients or caregivers, nor did they hold any line management or patient care responsibilities on the study ward. Patient and caregiver interviews took place on the ward, in the transit lounge, or via telephone (S1 Table). The interview guides were informed by the literature, our previous work related to unplanned hospital readmissions [6, 15, 16] and Eastern Health policies. The interviews with providers and patients occurred concurrently.

Staff interviews occurred on the ward near the time of patient discharge or before the end of their shift (S2 Table). Researchers determined data saturation by checking for repetitive content and no new information during observations and interviews. They confirmed data saturation by cross-checking observation notes and interview transcripts. The goal was to observe and interview a maximum of 30 discharges, but data saturation was reached after 21 patient and 42 staff interviews.

### Data analysis

Audio recordings of interviews were transcribed verbatim. Members of the research team validated these transcriptions. An inductive thematic analysis was done, following Braun and Clarke’s six-step framework [17]: Familiarisation with the data; generation of initial codes; exploration of emerging themes; review and refinement of identified themes; definition and naming of the final themes; and compilation of the final research report. Two researchers, namely OO and KWS, independently reviewed the transcripts to acquaint themselves with the data. They conducted separate coding, and subsequently collaborated to fine-tune the codes and develop and review the emerging themes through a consensus-building process. The research team collectively reviewed the transcripts and endorsed the identified themes. It’s important to note that an open coding approach was employed, allowing for the evolution and modification of codes during the coding process. This rigorous data analysis process assures the credibility (accurate and truthful representation of participants’ experiences), dependability, and trustworthiness of study findings [18]. However, no member checking, involving the return of transcripts to participants, was done. Data analysis was managed by NVIVO QRS10 software [19].

## Results

### Participant characteristics

Overall, 65 participants took part in this study. Twenty-one interviews were conducted with patients and two with caregivers. Forty-two staff (17 nurses, 10 doctors, 2 physiotherapists, 7 pharmacists, 1 social worker, 2 occupation therapists and 3 ward clerks) participated in interviews. The mean time for interviews conducted with patients and caregivers was 17 minutes. The mean time of interviews with health professionals was 20 minutes. The mean age of patients was 67 years and participant characteristics are summarised in Table 1.

**Table 1:** Participant characteristics.

**Patient Characteristics (N = 21)**	**Value**
Age, mean (SD)	67.85 (±17.94)
Sex (female), n (%)	11 (52.38)
Average length of stay	3.6 days
Diagnostic themes	
• Respiratory issues	n = 10
• Urinary issues	n = 4
• Gastrointestinal issues	n = 2
• Diabetes related issues	n = 2
• Falls	n = 1
• Other	n = 2
**Staff Characteristics (N = 42)**	
Medical	F = 6, M = 4
Nursing	F = 17
Occupation therapist	F = 2
Pharmacist	F = 6, M = 1
Physiotherapist	F = 2
Social Worker	F = 1
Ward Clerk	F = 3

### Semi structured interviews

**Fig 1:**
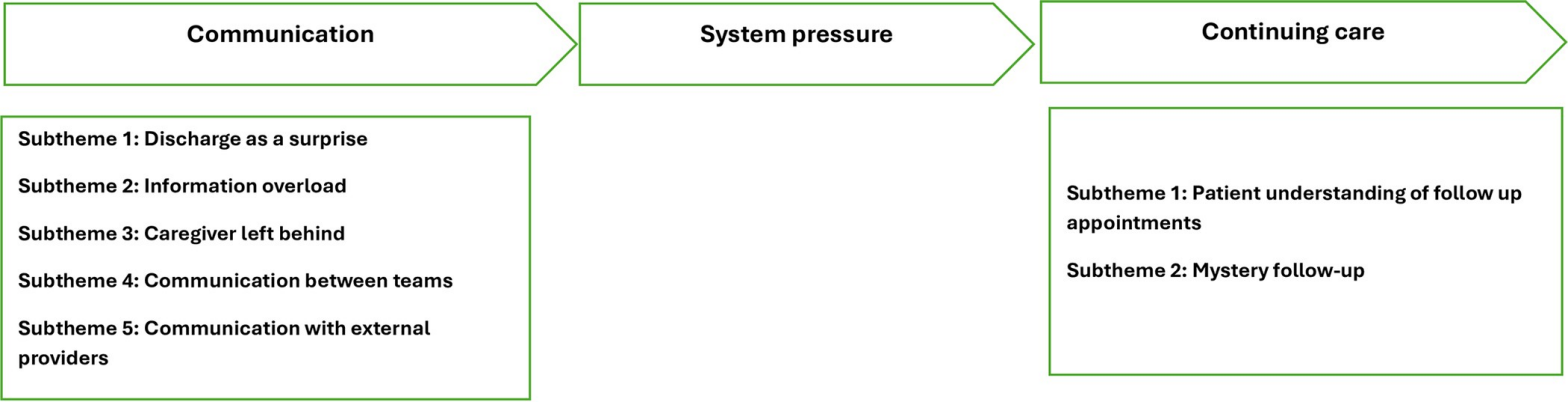
Key themes.

Three themes were identified from the data: ‘Communication’, ‘System Pressure’, and ‘Continuing care’ (Fig 1).

### Theme 1: Communication

The theme ‘Communication’ was by far the largest theme and reflected the way information is communicated, both between staff and patients (and their caregivers), and between staff. This theme had five sub-themes: i) discharge as a surprise, ii) information overload, iii) caregivers left behind, iv) communication between teams and v) communicating with external providers.

*Subtheme*: *Discharge as a surprise*. Discharge came as a surprise to patients, caregivers and staff. Patients described discharge being a short-notice event and lacking planning *‘‘…*. *I just don’t think maybe there’s enough notice … they told me at nine o’clock that I was going home*… *if my husband is to pick me up*, *that’s an hour’s drive*. *So maybe a little bit more notice for someone to come and pick me up…”* [Patient 1] *‘‘ So*, *I knew that I was going home just then*, *when I made the call to my partner*, *that’s when I knew that I was going home…at the same time*, *it would be nice to know you’re going to go home… I would have appreciated it if it was known earlier*, *then it ought to have been communicated earlier… she [patient partner] needed some notice*, *yeah absolutely*.*”* [Patient 2]

Staff also corroborated that “… *some of the discharges happen by surprise*.*”* [Clinician 1 (Pharmacist)] and that ‘surprise’ discharges create an additional stress for carers ‘‘.*… we’re giving them the call saying hey*, *they’re going home [laughs]*. *Surprise…*. *I think that’s what puts the stress on them*.*”* [Clinician 2 (Nurse)]. Clinicians suggested ways of forestalling surprise discharges, *‘‘I think one of the other things is maybe pre-empting or flagging that the patient is a potential discharge to the patient themselves*. *Perhaps earlier as well*. *Maybe the day before or a couple of days before*. *Yep*, *just so that they themselves (patients) are also prepared*.*”* [Clinician 3 (Doctor)].

*Sub-theme*: *Information overload*. Patients reported frustration about the amount of information given to them at the time of discharge’ *… the doctor came and … said… you can go home …*. *he was just telling me a whole lot of information…”* [Patient 2] with little time to clarify information being presented to them “… *I did ask a few questions … there’s too much information in too short a time*.*”* [Patient 2] Staff also acknowledged gaps in discharge information *‘‘*… *I don’t think we bother to like really to check their understanding …*. *actually*, *just checking their understanding I think is quite important as well*.*”* [Clinician 3 (Doctor)], and in particular patient’s understanding of discharge medications ‘‘…*they’re not able to tell me what they [medications] are for*, *but they can quite often tell me what the medication looks like and that they should be taking it at this time of day…”* [Clinician 4 (Doctor)]. Patients advocated for a discharge liaison person who can collate and convey all relevant information about their care and discharge plan in a streamlined manner and answer questions*‘‘ …the information that needs to be more streamlined…the idea of having some medical liaison*, *someone comes in and they’re like okay this is start and then they get pieces of all of the information and all of the different departments and comes in and says this is where we’re—to describe those kinds of processes*.*”* [Patient 3]

*Sub-theme*: *Caregivers left behind*. Caregivers felt they were not properly consulted ‘‘…*it would be good if the hospital were able to liaise with family… let us know what’s going on*… *I would like to hear from the doctors… what’s happening and where she [patient] is at and what… moving forward is going to mean and look like*.*”* [Caregiver 1] Staff also acknowledged suboptimal engagement with carers “…*when there’s a breakdown in communication… there’s not been a clear discharge plan… the family feel that… the patient has been chucked out and they’ve not got any more support…*” [Clinician 4 (Doctor)] and *“… sometimes the concerns of the family members aren’t being heard properly or investigated in depth enough…*.*”*[Ward Clerk 1]

*Sub-theme*: *Communication between teams*. Several problems arose concerning the communication of information about patient discharge. There was uncertainty about the day and the time of patient discharge by staff *‘‘…communication needs to be better…to be able to communicate to the patient with some more certainty… you’re going home today*…*”* [Clinician 5 (Nurse)] and patients “… *they sort of gave me an idea that I might be able to come home today but that’s been changing every day since I’ve been here …”* [Patient 4]. Staff commented on the suddenness of discharge notifications, “…*all of a sudden say*, *oh*, *they’re ready to go*. *We haven’t even called the family yet because we’re waiting to find out a time*. *So*, *I think they could communicate estimated times a bit better*.*”* [Clinician 6 (Nurse)]. Communication problems between teams were also recognised as a cause of delayed discharge, particularly related to preparation of discharge medications *‘‘…I think there could be some better communication around an estimated time that people’s medications will be ready… have a bit more of a standard… timeframe once they’ve got a script as to how long it will take*.*”* [Clinician 7 (Nurse)]. Conversely, receiving advanced notice of the discharge time hastened preparation of discharge medications ‘*‘…being notified in advance when someone’s definitely planning to go home is quite good because then we can… prep [prepare] that…”* [Clinician 1 (Pharmacist)].

Nursing staff felt excluded from the communication process employed by medical staff leaving them un-informed and unable to assist in disseminating information to patients *‘‘…us nurses aren’t aware… a lot of the time*, *patients are telling us they’ve been told they can go home when we haven’t been told that they’re allowed to go home yet… we would have no idea*.*”* [Clinician 8 (Nurse)]. Face to face communication was an important way of improving communication between physicians and other staff. Nurses commented that *‘‘…doctors talking to nurses probably a little bit more…*. *instead of just writing it in a note and putting the folder away…”* [Clinician 8 (Nurse)]. Allied health professionals also expressed a desire for “…*verbal communication to tell us as soon as you know when the patient is for discharge so we can prioritise them appropriately…”* [Clinician 9 (Physiotherapist)] but were also happy to use electronic tools “…*flag it… on the computer system*, *so we can know early and prioritise our day*.” [Clinician 9 (Physiotherapist)]

*Sub-theme*: *Communicating with external providers*. Staff felt that follow-up coordination and support after discharge is crucial and wanted better connection with external providers, particularly general practitioners (GPs), largely to compensate for limitations in current discharge summary processes. *‘‘… it would be useful to verbally contact the GPs when someone goes home… I know that a lot of GPs don’t receive discharge summaries in a timely manner… so*, *I think a verbal handover to the GP would be nice*. *Don’t think we have time to do it though*, *but that would be nice in theory*.*”* [Clinician 10 (Doctor)]. However, in some other cases communication with external providers is efficient *‘‘We also have really good communication with community pharmacies at the moment… technology is making it so that we can email through changes… I always call*, *they always receive it*, *so I’ve never had any communication issues*. *That always works pretty smoothly*.*”* [Clinician 11 (Pharmacist)]. To improve communication with external providers, ‘‘…*if the GPs could see on their system our letter (discharge summary) or if they could have access to our EMR thing*, *the electronic record*, *to see all the notes in there*, *that would help*. *That would be one thing*, *but I don’t know how feasible that is*.*”* [Clinician 12 (Doctor)].

### Theme 2: System pressure

The theme ‘System Pressure’ relates to the pressure to discharge patients. Staff perceived that there was pressure to discharge patients promptly to create bed availability *‘‘… they [management] just want to get people out of ED… they’re just looking at numbers rather than looking at the readmission rates… we’re under a lot of pressure … to get people out*.*”* [Clinician 12 (Doctor)] and that the need for bed availability could result in premature discharge “… *sometimes I think they were sent home too soon… there’s so much bed pressure at the moment”* [Clinician 13 (Doctor)]. Downstream, some staff felt that premature or rushed discharges resulted in unplanned readmissions *“*…*we’re not actually fully allowing patients time to recover or get to a point where they won’t be re-admitted*. *They’re going home quickly and then they’re coming back quickly*.*”* [Clinician 14 (Social worker)]. Others expressed concern about safety “*…I think things are … going to be missed because the pressure’s on and that’s not fair on the staff or the patient”* [Ward Clerk 1]. Pressure on staff was also exacerbated by limitations in service provision, particularly out-of-hours and on weekends, “…*weekends*… *we have limited pharmacy services… so*, *it just takes time for them to get their meds [medications]*.*”* [Clinician 15 (Doctor)]. Transport was also described as a pressure point “*If they’re going to go home by ambulance or patient transport… even though it’s been booked for a certain time… it ends up two to three hours later*.*”* [Clinician 15 (Doctor)].

### Theme 3: Continuing care

The theme ‘Continuing care’ described gaps in ongoing care and follow-up once patients left hospital and two sub-themes: i) Patient understanding of follow up appointments and ii) Mystery follow-up.

Sub-theme: Patient understanding of follow-up appointments. To ensure the continuity of their care after hospital discharge patients must have a clear understanding of their follow-up appointments. Some patients expressed difficulties in comprehending the follow-up appointments they themselves were to arrange and those that had been arranged for them to attend after leaving the hospital. For example, one patient was told their paperwork was sent to a specific hospital and ‘‘if you don’t hear from them, ring them… I don’t know when, how long I should wait before I ring them…” [Patient 2] and another patient expressed confusion about their follow-up ‘…’there’s one I’m not entirely sure, I need to call, I haven’t spoken to anyone about this … I don’t know what that’s about, thank you for reminding me because I don’t know what that’s about, I need to speak with someone who circled that and said call, but when? What is it for?” [Patient 3] When a caregiver was asked ‘do you understand any follow up appointments that she [patient] will need?’ they replied ‘‘… No, I don’t know what they are.” [Caregiver 1]. However, there were patients who did understand the process of scheduling and attending follow-up appointments “…I’ve got a couple of referrals… one for respiratory physio… and the other one … for heart hospital administration program, and they’ll be in contact regarding support at home…” [Patient 5].

*Sub-theme*: *Mystery follow-up*. The transition from the hospital to home can bring a sense of relief for patients, but it can also be a time of uncertainty and clinicians described not knowing what happened to patients once they left hospital. Staff highlighted that there was no way of knowing whether referrals were made “*… we might put a referral in… there is no ledger of who they’ve been referred to*.*”* [Clinician 10 (Doctor)] or whether patients accessed the services they were meant to once discharged from hospital, “*…we don’t have a very good way of tracking who is following the patient up once they go home… they might never see that endocrinologist or whatever …*.*”*. [Clinician 10 (Doctor)]. Staff acknowledged fragments in continuity of care *‘‘ … there is a bit of a disconnect between the primary health network and the hospital*.*”* [Clinician 10 (Doctor)], and gaps in home or community-based support ‘‘*the supports aren’t available in the community to support people in their home*, *so they’re coming into hospital*.*”* [Clinician 14 (Social worker)].

## Discussion

This study of hospital discharge processes for adult medical patients being discharged home from the perspectives of patient, caregiver and staff, identified three major process issues: i) variability in communication between clinicians, and between clinicians, patients and caregivers, ii) system pressures, and iii) patient and clinician uncertainty regarding follow-up.

Communication between teams during patient discharge in healthcare settings reveals critical challenges and opportunities for improving the coordination and quality of care. The findings in this study emphasise the importance of effective communication among healthcare professionals, to ensure smooth patient transitions from the hospital to home. One of the key challenges identified in the study is the uncertainty surrounding patient discharge. Clinicians and patients expressed concerns about the lack of clear and timely communication regarding the day and time of discharge. This uncertainty can cause stress and frustration for patients, as highlighted by the patient’s statement about continually changing discharge dates. Such inconsistencies in communication can hinder patients’ ability to plan for their return home, impacting their overall experience and satisfaction.

Clinicians also raised concerns about the suddenness of discharge notifications and the need for improved communication among the healthcare team. This abruptness can lead to unpreparedness on the part of nursing staff, pharmacists other allied health professionals, making it difficult to coordinate discharge logistics efficiently. Of note, the challenges in communication occurred despite standardised workflows designed to support inter-professional communication such as discharge planning and multidisciplinary team meetings. The lack of adequate notice about discharge plans for patients, caregivers, and even staff, as reflected in the findings, aligns with previous research indicating that unanticipated discharges can lead to increased stress and inadequate preparation for transition from hospital to home [20]. Patients and caregivers who are not adequately informed are less likely to understand post-discharge care instructions, increasing the risk of adverse events and readmissions [21]. At the time of this study the hospital maintained some pandemic requirements such as N95 masks for staff and limitations on visitors–both issues known to impact on communication [22, 23]. The impact of these factors–as well as emergence from an era of significant visitor restrictions and altered workflows caused by the pandemic–were not directly assessed by this study but may have influenced the findings. The need for better communication and coordination is a common issue in healthcare systems [24].

Nursing staff expressed frustration about feeling excluded from the communication process employed by medical staff, leading to a lack of awareness regarding patient discharge plans. Effective interprofessional communication is essential for providing high-quality care and ensuring patient safety [25]. The nurses’ desire for more face-to-face communication with physicians reflects the importance of direct interaction in clarifying patient status, addressing concerns, and fostering collaboration. Pharmacists and allied health professionals also highlighted the importance of early notification about patient discharge to prioritise their care plans and medication preparation and dispensing effectively. The communication challenges and solutions identified in this study align with international and Australian literature on healthcare communication and care coordination. Studies have consistently demonstrated the need for improved communication among healthcare teams to enhance patient safety, reduce delays, and improve the patient experience [26]. In Australia, the National Safety and Quality Health Service (NSQHS) Standards emphasise the importance of effective communication in healthcare settings, including during patient transitions [27]. The findings in this study reaffirm the relevance of these standards and the ongoing efforts to improve communication and coordination of care in Australian healthcare.

Furthermore, the role of the pharmacy team in providing services related to medications is crucial in the discharge process. The observed services, such as medication counselling, providing written information, and ensuring comprehension, align with best practices for medication management during discharge [28, 29]. However, the delay in notifying the pharmacy team about a patient’s impending discharge is a notable concern. Timely communication between healthcare providers is essential to prevent delays in the discharge process and ensure that necessary medications are ready when needed. Poor communication among healthcare team members can also lead to fragmented care and increased risk of errors [30].

Patients expressed frustration regarding the excessive amount of information provided to them at the time of discharge. They reported feeling overwhelmed and having little time to clarify the information presented. Such information overload leads to reduced patient satisfaction, and poor adherence to post-discharge instructions [31]. Furthermore, the observed variation in who informs family members or caregivers about the discharge process (patients, doctors, or nursing staff) highlights a potential communication gap in patient-centred care. Effective communication with family members and caregivers is crucial to ensure a smooth transition from the hospital to home [32]. Inconsistent communication practices can lead to misunderstandings, anxiety, and dissatisfaction among patients and their support networks. Further, suboptimal communication during hospital discharge is well-documented in the literature, with studies showing that patients often feel confused and unable to retain critical information provided to them during the discharge process [5].

In this study, healthcare staff, particularly clinicians, acknowledged the existence of gaps in discharge information. Clinicians recognised that they often do not take the time to ensure patients’ understanding of the information provided. This is a critical aspect of effective communication during care transitions [33]. Clinicians also noted that patients may not fully comprehend the purpose of their prescribed medications, highlighting the need for more patient-centred medication education during discharge [28]. The findings align with research emphasising the importance of patient education and shared decision-making in improving patient outcomes [34].

Study participants had a perception that swift patient discharges were prioritised to create inpatient bed availability to address issues like emergency department overcrowding and access block, and this was at the expense of the health and well-being of the patient being discharged. The pressure on efficiency was articulated by clinicians, who expressed concerns about the focus on numbers of discharges with little consideration of unplanned readmission rates. This echoes a prevalent issue in healthcare systems globally, where the emphasis on key performance indicators and bed occupancy rates can overshadow the importance of patient-centred care [35]. Similar situations have been observed in studies conducted in Europe, where concerns have been raised about discharging patients prematurely due to bed pressure [35–37] leading to increased readmissions and compromised patient outcomes. Paradoxically, hasty early discharge done to vacate inpatient beds to provide capacity for subsequent patients, may actually reduce hospital capacity as the rate of unplanned readmission is increased. The unplanned readmissions, that could potentially be prevented by better discharge processes, offset increased capacity obtained by hasty discharge processes. Improved discharge processes achieved without increased length of stay, could eliminate the waste and inconvenience of unplanned readmissions. Some improvement may be achieved by better practices using existing resources, but some may require increased staffing.

Patient understanding of follow-up appointments is a critical aspect of post-hospitalisation care and is essential for ensuring the continuity of care and positive health outcomes. The findings from this study shed light on the challenges and variations in patient comprehension of their follow-up appointments, which is consistent with existing literature both in Australia [38] and internationally [39]. The identified difficulties in comprehending follow-up appointments resonate with previous research conducted in the healthcare context. Patients’ lack of clarity about when and how to schedule follow-up appointments has been documented as a common issue [39]. The experiences shared by patients, who expressed uncertainty about when to contact the hospital if they didn’t receive communication, reflect a common challenge faced by discharged patients [40]. This highlights the need for healthcare providers to enhance communication and provide clear instructions regarding follow-up appointments, including appropriate timeframes for patient-initiated contact. Similarly, the response from caregivers, who reported not understanding the follow-up appointments, underscores the importance of not only educating patients but also involving their caregivers or support networks in the post-discharge care process. This aligns with recommendations from previous studies emphasising the role of caregivers in facilitating post-discharge care coordination [41].

The gap in home or community-based support, as highlighted in this study, is a common concern faced by healthcare professionals internationally [42]. The lack of adequate community-based resources can lead to readmissions, as patients may return to the hospital when they do not receive the necessary support and care at home [42]. This underscores the need for healthcare systems to strengthen community-based care options to facilitate a smoother transition from hospital to home. The issue of fragmented care is not unique to this study and has been identified as a significant challenge in healthcare systems globally [43]. The disconnection between hospitals and primary healthcare networks can lead to disjointed care delivery, where patients may not receive the continuity of care required for their recovery [44]. This disconnect can have adverse consequences for patient outcomes and healthcare efficiency [44]. Furthermore, the lack of a structured system to track referrals and patient follow-up, as expressed by clinicians, is a key concern. The absence of a centralised ledger or database for referrals and post-discharge care coordination can lead to missed opportunities for intervention and follow-up. Hence, there is a need for development of systems to track shared care plans to improve care continuity.

This study identified that power dynamics affected communication within teams, between patients and staff, and between staff and management. There were notable communication gaps between different healthcare teams, leading to uncertainty about discharge times and delays in preparing discharge medications. Nursing staff, in particular often learned about discharges from patients rather than directly from doctors, underscoring a hierarchical structure where doctors dominate communication, often neglecting to inform other team members in a timely manner [45, 46]. Healthcare organisations are deeply hierarchical in nature, with structures often based on authority or status derived from profession, expertise, gender, or ethnicity [47] and hierarchical structures and systems in healthcare organisations adversely impact on communication [47]. In this study, a patient- power staff dynamic with respect to communication was also evident. Patients and caregivers were often surprised by sudden discharge directives, reflecting a lack of involvement in decision-making. The lack of timely communication illustrates the dominant role of healthcare providers in making decisions without adequately consulting or informing patients and their families [48]. There was also a staff-management dynamic at play where staff felt pressured by management to expedite discharges, creating tension between operational efficiency and patient-centred care, and often leading to premature discharge and subsequent unplanned readmissions [36, 49].

### Key practical recommendations

The results of this study inform a number of practical recommendations for practice, policy and future research. First, systems should enable patients, caregivers, and staff to know about hospital discharge a day or two in advance. Second, streamlined information delivery via a discharge liaison role to collate and convey all relevant information in a clear and organised manner [50] would prevent information overload and ensure that patients and caregivers fully understand the discharge plan and follow-up care. Third, caregiver involvement needs to be improved with protocols and procedures for regular caregiver updates to ensure caregivers are well-informed and prepared for the patient’s discharge, enhancing support and care continuity at home. Fourth, enhanced inter-professional communication through regular interdisciplinary meetings and use of shared electronic health records. Firth, clear follow-up instructions by providing patients and carers with detailed, easy-to-understand follow-up schedule, including contact information for arranging appointments. Finally, effective external provider coordination through development of standardised processes for communicating discharge summaries to general practitioners and other external providers promptly.

### Strengths and Limitations

The study’s strength lay in its rigorous qualitative methodology involving collection of data from both patients/caregivers and staff. This approach provided a platform for patients/caregivers, and staff to express their experiences, perspectives, and opinions. Notably, the interviews occurred immediately post-discharge, mitigating recall bias and enabling the sharing of fresh, unfiltered experiences. However, it’s important to acknowledge certain limitations in interpreting the study’s findings. The interviewed participants primarily consisted of English-speaking individuals who returned home and had no cognitive impairment. To comprehensively capture patient experiences, future research should prioritise including residents in care facilities and those with limited English proficiency. Additionally, the study was conducted during COVID-19 visitor restrictions, which hindered access to caregivers.

## Conclusion

This study identified gaps relating to hospital discharge process despite relatively mature system measures to support synchronous inter-professional communication. Notably, some of these measures (multidisciplinary team meetings and discharge planning huddles) do not include patients, emphasising a need for asynchronous communication tools that can be challenging to implement in the environment of a hybrid medical record such as the study hospital. This has implications for patient experience of health care and patient safety, and clinician work satisfaction and engagement. Effective communication, especially among different healthcare professionals, emerged as a critical factor in ensuring smooth transitions from hospital to home. Addressing uncertainties and information overload for patients and caregivers is vital for enhancing patient satisfaction and adherence to post-discharge instructions. Roles such as discharge ambassadors may be useful in bridging communication gaps between clinicians and patients to minimise these identified gaps. There is a critical need to design and test interventions that improve community-based support, increase integration of acute hospital care and post-hospital care (such as GPs / primary care) and systems to enable tracking referrals and follow-up care, to ensure safe hospital discharge whilst optimising patient, caregiver, and staff experience of the discharge process.

## Supporting information

S1 Table Final interview guide patient/caregiver. (DOCX)

S2 Table Final interview guide staff. (DOCX)
