# Supplementary material for: Hospital discharge processes: Insights from patients, caregivers, and staff in an Australian healthcare setting
Source: PLoS One. 2024 Sep 19;19(9):e0308042. doi: 10.1371/journal.pone.0308042 (PMC11412517; doi:10.1371/journal.pone.0308042)
Supplement: S1 Table — (DOCX) [file pone.0308042.s001.docx]

## S1 Table: Interview Guide for Patients / Caregivers

| Example questions (will be tailored to patient or carer) |
| --- |
| - Have you had many admissions to hospital in the last 6 months? - When did you find out you were going home? - How ready do you feel to go home? - What are you looking forward to the most about going home? - Do you feel like you have enough information about your condition to manage at home? - Do you feel you understand which medications you need to take at home, what they are for and when you need to take them? - How are you getting home? - Do you have someone that helps you once you get home? - Do you understand what follow-up appointments you will need to have once you go home? - Have you been told that there are activities you cannot do once you are at home? - Do you have any other comments about the process of going home from hospital? If there was one thing we could improve, what would it be? |
