# Supplementary material for: Hospital discharge processes: Insights from patients, caregivers, and staff in an Australian healthcare setting
Source: PLoS One. 2024 Sep 19;19(9):e0308042. doi: 10.1371/journal.pone.0308042 (PMC11412517; doi:10.1371/journal.pone.0308042)
Supplement: S2 Table — (DOCX) [file pone.0308042.s002.docx]

S2 Table: Interview Guide for Staff

| Example questions (will be tailored to staff role) |
| --- |
| - Patient [name] was discharged [insert day]. Can you tell me how the decision for discharge was made? - Once the decision is made that someone is ready to go home, how is that information conveyed? - If a patient or carer says they are not ready for discharge, how is this managed? - Do you feel like you have a strong sense of how patients / carers will manage at home? - Do you feel that patients / carers understand the medications they need to take at home and what they are for? - Can you tell us about the process for the patient to actually leave the ward? - Do patients / carers ever contact the ward once they are at home? - In your experience, do many patients experience an unplanned hospital readmission soon after they go home? - Do you have any other comments about the process of going home from hospital? If there was one thing we could improve, what would it be? |
